# Comparative Degradome Analysis of the Bovine Piroplasmid Pathogens *Babesia bovis* and *Theileria annulata*

**DOI:** 10.3390/pathogens12020237

**Published:** 2023-02-02

**Authors:** Tomás Javier Poklepovich, Maria Mesplet, Romina Gallenti, Monica Florin-Christensen, Leonhard Schnittger

**Affiliations:** 1 Instituto de Patobiología Veterinaria (IPVET, INTA-CONICET), Centro de Investigaciones en Ciencias Veterinarias y Agronómicas (CICVyA), Instituto Nacional de Tecnología Agropecuaria (INTA), Los Reseros y Nicolas Repetto s/n, Buenos Aires 1686, Argentina; 2 Consejo Nacional de Investigaciones Científicas y Técnicas (CONICET), Buenos Aires 1033, Argentina

**Keywords:** *Theileria annulata*, *Babesia bovis*, bovine babesiosis, tropical theileriosis, peptidases, proteinase repertoire, degradome, comparative degradomics

## Abstract

*Babesia bovis* and *Theileria annulata* are tick-borne hemoprotozoans that impact bovine health and are responsible for considerable fatalities in tropical and subtropical regions around the world. Both pathogens infect the same vertebrate host, are closely related, and contain similar-sized genomes; however, they differ in invertebrate host specificity, absence vs. presence of a schizont stage, erythrocyte invasion mechanism, and transovarial vs. transstadial transmission. Phylogenetic analysis and bidirectional best hit (BBH) identified a similar number of aspartic, metallo, and threonine proteinases and nonproteinase homologs. In contrast, a considerably increased number of S54 serine rhomboid proteinases and S9 nonproteinase homologs were identified in *B. bovis*, whereas C1A cysteine proteinases and A1 aspartic nonproteinase homologs were found to be expanded in *T. annulata*. Furthermore, a single proteinase of families S8 (subtilisin-like protein) and C12 (ubiquitin carboxyl-terminal hydrolase), as well as four nonproteinase homologs, one with dual domains M23-M23 and three with S9-S9, were exclusively present in *B. bovis*. Finally, a pronounced difference in species-specific ancillary domains was observed between both species. We hypothesize that the observed degradome differences represent functional correlates of the dissimilar life history features of *B. bovis* and *T. annulata*. The presented improved classification of piroplasmid proteinases will facilitate an informed choice for future in-depth functional studies.

## 1. Introduction

*Babesia bovis* and *Theileria annulata* are tick-borne hemoprotozoans that restrain livestock industry and result in significant economic losses in tropical and subtropical regions around the world [1,2,3,4,5]. They belong to closely related phylogenetic lineages, infect the same vertebrate host, and display many comparable biological characteristics, including a similar-sized 8.2 Mb genome comprised of four chromosomes [6,7,8,9,10].

*B. bovis* causes bovine babesiosis or redwater fever in cattle, characterized by severe anemia and nervous signs due to sequestration of infected red blood cells in the brain capillaries, and can be fatal [4,5]. Its life cycle is initiated by tick-transmission of the infective sporozoite stage into the bovine blood. Sporozoites invade erythrocytes and develop into the merozoite or piroplasmid stage. Merozoites egress after duplication, invade new erythrocytes, continuing their asexual intraerythrocytic duplication or develop into micro and macrogamonts. Upon a tick blood meal on an infected bovine, the parasite forms sexual stages within tick tissues and finally develops into infective sporozoites in the salivary glands [9,10,11,12,13]. The main tick vector is *Rhipicephalus microplus*, ubiquitously distributed in subtropical and tropical regions, yet some other species of *Rhipicephalus* function also as competent vectors in some regions [4,9,14]. Importantly, *B. bovis* belongs to the phylogenetic lineage of *Babesia* sensu stricto (s.s.) parasites (“the true *Babesia*” defined as Clade VI [8,9]). This group of *Babesia* parasites is characterized by transovarial transmission, and is passed on in a vertical mode to the following tick generation [9,11,13,14,15].

*T. annulata* causes tropical theileriosis of cattle, characterized by fever, anemia, and lymph node inflammation. In contrast to *B. bovis*, *T. annulata* is exclusively endemic in the tropical and subtropical regions of North Africa, the Mediterranean region, the Middle East and Asia, but not in the Americas [9,16,17]. A distinguishing feature of the *T. annulata* life cycle compared to that of *B. bovis* is that sporozoites invade macrophages and/or B lymphocytes to develop into the schizont parasite stage, where they produce the malignant transformation of host cells, resulting in an often-fatal leukoproliferative disease [18,19]. This characteristic is unique among protozoans and is confined to the subclade of ‘transforming *Theileria’* that places within the phylogenetic lineage of *Theileria* s.s. parasites (Clade V [8,9]). *Theileria annulata* is transmitted among ticks in a transstadial fashion. In this mode of transmission, the pathogen is acquired by the feeding larvae or nymph and is transmitted to the vertebrate host by the succeeding nymph or adult tick stage, respectively [10,11,12,13]. Following feeding, the transmitting tick stage is cured of the infection, resulting in a horizontal transfer of *Theileria* parasites in the tick population. Tick species of the genus *Hyalomma* spp. represent the main vectors of *T. annulata* [20]. 

On one hand, *B. bovis* and *T. annulata* show major similarities, such as genome size, chromosome count, vertebrate host specificity, and intraerythrocytic duplication in the mammalian host, sexual reproduction in the tick gut and infective sporozoite formation in tick salivary glands [9,13]. On the other hand, they differ in at least four principal characteristics: (i) invertebrate host specificity (transmission by *Rhipicephalus* vs. *Hyalomma* spp.), (ii) mode of tick transmission (transovarial vs. transstadial transmission), (iii) parasite stages (absence vs. presence of the schizont parasite stage), and (iv) erythrocyte invasion mechanism (transient formation of a parasitophorous vacuole vs. zipper mechanism) [21].

Due to the involvement of parasite proteinases in vital functions, and given that many show low or no identity with host encoded proteinases, proteolytic enzymes have been proposed as potential drug targets and/or as vaccine candidates [22,23,24,25,26,27,28,29]. A proteolytic enzyme that is a hydrolase is also known as a proteinase, protease, or a peptidase. In principle, these are synonyms but to avoid confusion the term proteinase is preferably used here [30]. Proteinases have been shown to be involved in many diverse and complex cellular processes, such as growth and metabolism, cell cycle regulation, host-cell adhesion and host-cell invasion and egress in related Apicomplexa, such as *Plasmodium falciparum* [31,32]. As host cell invasion and egress are obligatory steps in the life cycle of apicomplexan parasites, proteinases involved in these processes are potential targets for therapeutic interventions [33]. For example, the serine proteinases subtilisin-like peptidase 1 (SUB1) and subtilisin-like peptidase 2 (SUB2) of *P. falciparum* play a key role in host cell invasion and egress, respectively [34,35,36]. Importantly, SUB1 and SUB2 are processed and activated by the aspartic proteinase plasmepsin X, while plasmepsin IX is involved in parasite development within the parasitophorous vacuole (PV) and oocyst formation. The corresponding aspartic protease orthologs ASP3b and ASP3a of *B. bovis* and *T. annulata* have been proposed to be involved in homologous processes [37]. Moreover, parasite rhomboid proteinases have been described to participate in adhesion to and internalize into the host cell [38,39,40,41]. Importantly, two potent *P. falciparum*-specific ROM4 inhibitors designated rhomboid-inhibiting ketoamide (RiKa) and rhomboid-inhibiting boronate (RiBn) have been recently developed [42,43,44].

In addition, parasite egress, an obligatory step in parasite propagation, and degradation of host proteins, such as hemoglobin, are mediated in *Plasmodium* sp. by parasite cysteine proteases [45,46,47,48,49,50]. In accordance with potential similar roles in piroplasmids, cysteine proteinase inhibitors have been shown to impede in vitro the parasite growth of several *Babesia* species and *T. equi* [51]. Furthermore, it has been proposed that proteinases involved in parasite egress, signal peptide processing and protein secretion may be good candidates for antimalarial targeting, as they have been shown to be highly connected in protein networks [32]. Proteinases involved in processing and the catabolism of proteins have been also studied. Thus, the use of bestatin, as an inhibitor of leucine aminopeptidase, involved in the processing, catabolism, and degradation of intracellular proteins, was tested, and shown to inhibit moderately in vitro growth of *B. bovis* [52]. Furthermore, inhibition of methionine aminopeptidase, involved in intracellular protein processing, resulted in significant inhibition of in vitro and in vivo growth of *B. bovis* [53]. However, degradation of hemoglobin, the main available protein for piroplasmid nutrition inside erythrocytes, has not been experimentally established and needs further exploration [54].

Previously, we identified 66 and 64 proteinases predicted as active in the proteomes of *B. bovis* and *B. microti* [55,56]. Comparison of both arrays of proteinases showed that a representative of family S8 is present in *B. bovis*, but not in *B. microti*, whereas a representative of family A22 is not present in *B. bovis*, but in *B. microti* [56]. These differences may be related to the different evolutionary history of *Babesia* s.s and *Babesia* sensu lato (s.l.). 

In the present study, we have thoroughly compiled and compared the proteinase repertoires of *B. bovis* and *T. annulata* bovine pathogens using bioinformatic tools. In addition, the presence of signal peptides, transmembrane regions, and ancillary domains has been analyzed, allowing insight into the cellular location and potential function of individual proteinases. This analysis can facilitate in the future the rational selection of proteinases as vaccine candidates and/or drug targets. Furthermore, we compare and highlight the differences in the proteinase repertoire between both bovine pathogens, as they may represent potential functional correlates of their individual life cycle history.

## 2. Results

### 2.1. Total Degradome Proteinase Repertoire

The degradome of *B. bovis* has been determined to consist of 133 proteinases and nonproteinase homologs, comprising 82 functional and 51 non-functional proteinases (Table 1). Thus, the degradome corresponds to approximately 3.5% of the proteome of *B. bovis*. Noteworthy, 72 (35 functional and 37 non-functional proteinases) of the identified 133 proteinases have not yet been recognized by the MEROPS database (October 2022). A comparable number of 132 proteinases and nonproteinase homologs were identified for *T. annulata*, of which 80 represent functional and 52 non-functional homologs. Among the 132 proteinases, 18 proteases (four functional and 14 non-functional) have not yet been recognized by the MEROPS database (October 2022). Similar to *B. bovis*, the proteinase repertoire accounts for 3.3% of the total *T. annulata* proteome (Table S1). 

In both pathogens, aspartic and threonine proteinases were less abundant than other types, with six and 14 proteinases in *B. bovis*, respectively, and eight and 15 proteinases in *T. annulata*, respectively (Table 1). In contrast, cysteine, metallo, and serine proteinases are at least twice as frequent, comprising 26, 36, and 51 in *B. bovis*, and 36, 35, and 38 in *T. annulata*, respectively. Major differences are observed for cysteine proteinases, of which *B. bovis* encodes only 26, against 36 in *T. annulata*, as well as for serine proteinases, of which *B. bovis* encodes 51, against only 38 in *T. annulata*. Thus, the *T. annulata* degradome includes 10 additional cysteine proteinases with respect to that of *B. bovis*, while *B. bovis* encodes 13 additional serine proteinases, compared to *T. annulata*, resulting in a similar total proteinase repertoire of both pathogens.

### 2.2. Orthologous and Species-Specific Proteinases

Altogether, 108 ortholog pairs, as determined by BBH, were identified of which in 54 pairs, both proteinases were found to be functional. A further 48 were composed of two non-functional proteinase homologs and the remaining six pairs contained a functional proteinase and a nonproteinase homolog (Table 2(a)).

Twenty-five species-specific proteinases were identified in *B. bovis*, of which seven represent functional proteinases and 18 non-functional proteinase homologs. Correspondingly, 24 species-specific proteinases could be identified in *T. annulata*, of which 14 represent functional proteinases, and 10 non-functional homologs (Table 2(b)).

### 2.3. Proteinase Clans and Families

The degradomes of *B. bovis* and *T. annulata* were organized into classes, clans, and families (Figure S1). The number of proteinases assigned to each category is shown, as well as the number of functional vs. non-functional proteinases. Proteinases of *B. bovis* could be allocated to 40 different families that grouped into 26 clans, while proteinases of *T. annulata* are represented by 38 families that are distributed to 25 different clans. Both piroplasmid pathogens encode two aspartic, one threonine, and 13 metalloproteinase families, which correspond to one, one, and 10 clans, respectively. Noteworthy, a single metalloproteinase of each piroplasmid species represents a member of metalloproteinase family M79, yet this family has been recently redesignated into the glutamic proteinase family G5; however this needs experimental confirmation. Cysteine proteinases represent 15 families (5 clans) in *B. bovis*, but since family C12 (ubiquitin carboxyl-terminal hydrolase) is absent in *T. annulata*, only 14 families (six clans) of cysteine proteases are present in the latter parasite species. Serine proteinases of *B. bovis* and *T. annulata* consist of 10 and nine families, corresponding to nine and eight clans, respectively; since family S8 (subtilisin-like protein), which is the only member of clan SB, is absent in *T. annulata*. In summary, the observed differences between both pathogens can be attributed to the lack of cysteine proteinase family C12 and serine proteinase family S8 of clan SB in *T. annulata*. The proportion of functional vs. non-functional proteinases among proteinase types is relatively constant between both species, with the exception that only 20 functional cysteine proteinases are encoded by *B. bovis*, but 29 in *T. annulata*, whereas 33 functional serine proteinases are encoded by *B. bovis*, but only 24 in *T. annulata* (Figure S1).

### 2.4. Proteinases with Two Proteinase Domains

Notably, proteinases bearing two proteinase domains of the metalloproteinase families M16 and M23, and serine proteinase family S9 were identified in the *B. bovis* degradome. In each case, the two domains where designated as upstream (a) and downstream domains (b) (Table 3 and Table S1). Proteinases BBOV_III003850 and BBOV_IV001260 were each found to be composed of two M16 domains. In the former, both encoded domains are non-functional, while the latter encodes a functional and a non-functional domain. In addition, proteinase BBOV_II001730 exhibits two non-functional domains of the M23 family. Furthermore, proteinases BBOV_III011180, BBOV_IV003330, and BBOV_II003080 were each found to be composed of two S9 proteinases, of which none is functional. Unlike *B. bovis*, *T. annulata* features the two proteinases TA11975 and TA19130, which each present dual M16 domains. Both domains encoded by TA11975 are non-functional homologs, while TA19130 encodes a proteinase comprising of a functional and a non-functional domain.

### 2.5. Molecular Phylogenetic Tree of the Proteinase Domains

**Figure 1:**
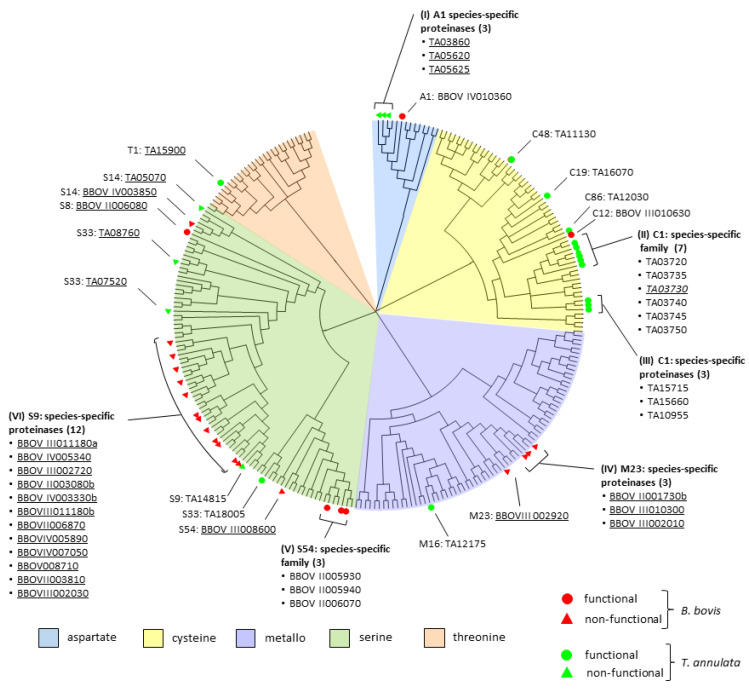
Global phylogenetic proteinase wheel of proteinase and nonproteinase homolog domains of *B. bovis* and *T. annulata*. The global tree was generated using the protease domain from one member of each catalytic type, and individual family trees were added at the corresponding positions. Colors indicate proteinases of different catalytic types: blue, aspartic type; light yellow, cysteine type; light violet, metallo type; green, serine type, and light brown, threonine type. Species-specific functional proteinases (circles) or nonproteinase homologs (triangles) of *B. bovis* (red) and *T. annulata* (green) are designated with their corresponding gene ID. Underlined gene IDs refer to nonproteinase homologs. Clockwise, roman numerals I to VI indicate species-specific paralog families of proteinases and nonproteinase homologs. Remaining designations indicate single species-specific proteinases or nonproteinase homologs. The designation of the proteinase family (MEROPS) is given before the gene ID of *B. bovis* and *T. annulata* proteinases and nonproteinase homologs.

A global phylogenetic tree was constructed comprising all proteinases and nonproteinase homologs of both species (Figure 1). The global tree shows the distribution of proteinases and nonproteinase homologs into different classes. Furthermore, the tree allows for confirmation of pairs of proteinase orthologs, identification of species-specific proteinases or of families of proteinase paralogs in *B. bovis* or in *T. annulata*, respectively. In *T. annulata*, three groups of species-specific proteinase families could be identified and are represented by three non-functional proteinases of family A1 (I), and a group of six (II) and another of three functional proteinases (III), each of which belong to the C1A family.

In *B. bovis*, groups of species-specific proteinase families are represented by three non-functional proteinases of the family M23 (IV), three functional proteinases of the family S54 (V), and 12 non-functional proteinase homologs of the family S9 (VI).

Of these proteinase paralog families, exclusively the species-specific proteinase paralog family S54 in *B. bovis* (V), and the two families comprising six (II) and three C1A proteinases (III) in *T. annulata* represent functional proteinases (Table 4). In addition, single species-specific functional proteinases belonging to families A1, C12, C97, S8, and S9 that were identified in *B. bovis*, and of families C19, C48, C86, C88, M16, S33, S54, and T1 identified in *T. annulata* are listed in Table 4. Notably, the largest differences in the number of species-specific functional proteinases between both pathogens is observed for the cysteine and the serine types: *B. bovis* presents only two species-specific cysteine but five serine proteinases, of which three belong to the S54 family. In contrast, *T. annulata* encodes 12 species-specific cysteine, of which eight belong to the C1A family, but only two serine proteinases. Nonfunctional species-specific single proteinases constitute BBOVIII002920 (M23), BBOVIII008600 (S54), and BBOVIV003850 (S14) for *B. bovis* and TA03730 (C1), TA14815 (S9), TA07520 (S33), TA08760 (S33), and TA05070 (S14) for *T. annulata* and are designated in the tree (Figure 1).

### 2.6. Secreted and Membrane Proteinases

The prediction of a signal peptide, membrane topology, and extracellular vs. intracellular localization was determined for both degradomes (Table S2). Of the total degradome of 133 peptidases of *B. bovis*, 28 were identified to be secreted (*n* = 9) or represent membrane proteinases (*n* = 19) that are either single-pass (*n* = 3) or multitopic membrane proteins (*n* = 16). A higher number of 36 proteinases of the *T. annulata* degradome (*n* = 132) are secreted (*n* = 11) or membrane associated (*n* = 25), either as single-pass (*n* = 12) or as multitopic membrane proteins (*n* = 13). Remaining proteinases of both degradomes are not listed in Table S2 and represent intracellular cytoplasmic proteinases. Each class of proteinases includes members that are characteristic for their cellular location and/or membrane topology. Thus, all aspartic A1 proteinases are secreted into the extracellular matrix. In contrast, nearly all single-pass proteinases belong to the cysteine proteinases. That is, two C1A cysteine proteinases of *B. bovis* and 10 of *T. annulata* represent single-pass type II membrane proteins. The largely increased number of this type of proteins in the latter pathogen can be explained by the above-mentioned expansion of C1A proteinases in *T. annulate,* compared to *B. bovis.* Interestingly, the exclusive example of a type I protein is a C13 proteinase of *B. bovis*, while the corresponding ortholog of *T. annulata* is predicted to be secreted. Furthermore, all multitopic proteinases are found to belong either to metalloproteinase or to serine proteinases. Of metalloproteinases, only *T. annulata* encodes a M23 proteinase predicted to be secreted, while in both species metalloproteinases of family M41 exhibit either one or two transmembrane domains, or represent multi-pass proteins with six or seven (family M48), or even nine transmembrane domains (family M79). Of the serine proteinases, only a single S8 family member predicted to be secreted was identified in *B. bovis* and a single member of the S9 family was identified as a multitopic proteinase homolog in *T. annulata*. In addition, each member of an orthologous pair of S26 proteinases has two transmembrane domains. Remaining proteinases belong to the multitopic rhomboid S54 family with two to seven transmembrane domains. Corresponding to the above-mentioned expansion of S54 proteinases in *B. bovis*, an increased number of ten S54 proteinases were identified in this parasite, compared to only six in *T. annulata*. Interestingly, all proteinases of the threonine class are intracellular proteinases and none was identified to be secreted or represent a membrane proteinase.

### 2.7. Ancillary Domains

**Figure 2:**
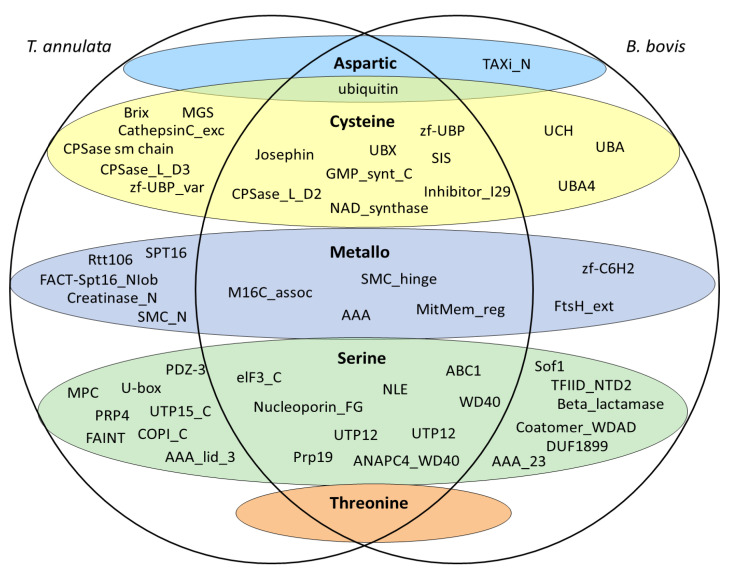
Ancillary domains present in proteinases and nonproteinase homologs of *B. bovis* (right circle) and *T. annulata* (left circle). Each proteinase sequence was analyzed by Pfam (InterPro) for the presence of nonproteinase domains. Ancillary domains are displayed with their short name according to Pfam.

The degradomes of *B. bovis* and *T. annulata* were analyzed with respect to proteinase-associated ancillary domains. The distribution of ancillary domains in proteinase classes and both piroplasmid species is displayed in a Venn diagram (Figure 2). Altogether, 51 of 132 proteinases and nonproteinase homologs (38.6%) of *T. annulata* contain one to multiple (up to five) ancillary domains (e.g., the cysteine proteinase TA02545 contains five ancillary domains: GMP_synt_C; MGS; NAD_synthase; CPSase_L_D2; CPSase_L_D3. A similar number of 53 of the 133 proteinases and nonproteinase homologs (39.8%) of *B. bovis* was associated with at least one ancillary function.

Interestingly, only two different ancillary domains were found to be associated with aspartic and none with threonine proteinases. In contrast, the majority of ancillary domains were linked with cysteine proteinases (*n* = 18), metalloproteinases (*n* = 11), and in particular with serine proteinases (*n* = 24). Remarkably, 22 of a total of 52 ancillary domains are shared by both piroplasmids while 30 were identified as species-specific. Of the latter, a higher number was identified in proteinases and nonproteinase homologs of *T. annulata* (*n* = 18), compared to *B. bovis* (*n* = 12). Interestingly, the ancillary domain WA40 was identified 25 times in the degradome of *B. bovis* but only 15 times in that of *T. annulata* (Table S1).

## 3. Discussion

Proteolytic enzymes are a group of highly diverse proteins that can cleave a carbon-nitrogen bond between two amino acids. Most proteolytic enzymes are proteinases that hydrolyze a peptide bond by activating a water molecule. According to the composition of their nucleophile, they belong to the class of aspartic, cysteine, metallo, serine, threonine, and glutamic proteases. Additionally, a small group of proteolytic enzymes, known as asparagine lyases, cleaves the peptide bond by cyclization of an asparagine residue to a succinimide [57]. The present work shows that the genomes of *B. bovis* and *T. annulata* encode representatives of the former five protease classes; however, metalloproteinase family 79 has been recently redesignated as glutamic proteinase family G5. Although, this redesignation has not yet been experimentally confirmed, it suggests that a single glutamic proteinase is encoded by each piroplasmid species. The corresponding putative glutamic proteinase ortholog is found as well in *P. falciparum*, one of the model organisms in the MEROPS database, which belongs, together with Piroplasmida, to the Aconoidasida class of the phylum Apicomplexa. Moreover, *Toxoplasma gondii*, an apicomplexan protozoon of the Conoidasida class, contains two glutamic proteases, of which one is an ortholog of the glutamic proteinase found in Aconoidasida. Asparagine lyases have not been found so far in any studied Apicomplexa [58].

Proteinases are present in all organisms, suggesting they must have emerged at the earliest stages of protein evolution as degradative enzymes necessary for protein catabolism and the generation of amino acids in primitive organisms. However, beyond these nonspecific degradative functions, proteinases act as sharp scissors and catalyse highly specific reactions of proteolytic processing that regulate fate, localization, and the activity of many proteins, modulate protein-protein interactions, create new bioactive molecules, contribute to the processing of cellular information, and generate, transduce, and amplify molecular signals [59].

Apart of proteinases with proteolytic activity, a large number of proteinase families (about 70% of proteinase families defined by MEROPs) include inactive proteinase homologs (also referred to as nonproteinase homologs or pseudoproteinases) and it has been concluded that these inactive proteinase homologs have evolved from their active counterparts [60,61,62]. Originally, it had been assumed that nonproteinase homologs exert only regulatory functions through competitive inhibition. However, meanwhile it has been established that they have evolved additional multiple functions that are of vital importance and are often associated with regulatory activities, such as signal transduction [63,64,65]. In this study, we refer to the complete reservoir of active proteases and nonproteinase homologs encoded by a single species as the degradome.

The present work aims to analyze degradome differences between the well-studied and closely related *B. bovis* and *T. annulata* piroplasmids. As both these piroplasmid species share many biological characteristics, life history differences may cautiously be attributed to differences in the degradome composition and structure. We found that a similar percentage of 3.5% and 3.3% of the proteome correspond to proteinases and nonproteinase homologs of *B. bovis* (*n* = 133) and *T. annulata* (*n* = 132), respectively. In addition, a similar proportion of active vs. inactive proteinases was found to be encoded in the genome of *B. bovis* (82 of 133, 61.7 % vs. 51 of 133, 38.7%) and *T. annulata* (80 of 132, 60.6% vs. 52 of 132, 39.4%), respectively. When analyzing the different proteinase classes, a similar number of aspartic, metallo, and threonine proteinases and nonproteinase homologs was identified in each parasite. In contrast, significant differences were observed in the number of cysteine and serine proteinases. Thus, a substantially decreased number of 10 cysteine proteinases are encoded by *B. bovis* (*n* = 26), compared to *T. annulata* (*n* = 36), whereas 13 additional serine proteinases are encoded by *B. bovis* (*n* = 51), compared to *T. annulata* (*n* = 38). 

Molecular phylogeny of proteinase domains allows for identifying orthologous pairs of proteinase domains, expansion and/or loss of proteinase families, and species-specific proteinase domains. Orthology can either be determined by phylogeny or BBH [66]. Phylogenetic approaches are considered more reliable but are limited to proteinases with near-complete domain sequences that can be aligned. We found in this study that whenever the nearly complete domain sequence was available, molecular phylogeny and BBH led to the same result.

By definition, orthologs arose through an evolutionary speciation event and therefore have the same function [66]. Accordingly, it can be assumed that the 108 orthologous proteinase pairs identified are responsible for basic metabolic functions shared by both piroplasmids. Interestingly, the large majority of orthologous pairs are either composed of functional (*n* = 54) or non-functional orthologs (*n* = 48). The remaining six pairs are composed of a functional and non-functional proteinase, suggesting that nucleotide exchanges at enzymatically active sites succeeded gene duplication and are more recent events.

It is reasonable to assume that species-specific proteinases most likely correspond with functions that are specific/characteristic for each species. Interestingly, of the 25 species-specific proteinases identified in *B. bovis*, a large majority of 18 belong to serine proteinases, while of the 24 species-specific proteinases identified in *T. annulata*, a large number of 12 represent cysteine proteinases (Table 2(b)). 

More detailed molecular phylogenetic analysis suggests that the observed differences in the repertoire of cysteine and serine proteinases is mainly due to a substantial expansion (or loss) of members of the enzymatically active C1 and S54 family of *T. annulata* and *B. bovis*, respectively. In contrast, expanded numbers of non-functional proteinase domains were identified in proteinase families A1 and M23/S9 in *T. annulata* and *B. bovis*, respectively. Thus, serine and cysteine proteinases, in particular, may play an important role in the unique biological characteristics associated with each of both piroplasmid species.

The presence of certain protease families in all kingdoms of life (bacteria, archaea, plants, animals, fungi, protozoa ,and chromists) has substantiated the hypothesis that they were already present in the last universal common ancestor (LUCA). Of the 33 families believed to have this ancient origin, 19 are present in *B. bovis* and *T. annulata* and include the cysteine proteases of families C26, C44, and C56; the metallo proteases of families M1, M3, M16, M24, M38, M48, M67, and M79; the serine proteases of families S1, S9, S12, S16, S26, S33, and S54, and the threonine proteases of family T1 [57].

Among these ancient proteinase families, we have recently identified members of the S54 family, or rhomboid proteases, in ten different piroplasmid species belonging to the genera *Babesia*, *Theileria* and *Cytauxzoon* for which whole genome sequences are available [67]. Rhomboid proteases are particularly intriguing since they are membrane-bound endoproteinases with their active site embedded within the lipid bilayer. They cleave peptidic bonds within or adjacent to a single pass transmembrane domain of their protein substrate. In *P. falciparum* and *T. gondii*, some rhomboids have been shown to cleave parasite adhesins, dismantling the tight junctions formed between parasite and host membranes during the invasion process, thus allowing the parasite to be internalized into the host cell [68]. Consistent with an essential role in parasite invasion, rhomboid inhibitors specifically hamper the in vitro growth of *P. falciparum* [44]. Piroplasmid rhomboids belong to the ROM4, ROM6, ROM7, and ROM8 groups, and whereas in the latter three a single representative is present in the studied piroplasmid species, ROM4 has two representatives in *Theileria* s.s., *T. equi*, *B. microti*, and *Cytauxzoon felis*, but three or more in *Babesia* s.s. Specifically, in *B. bovis,* the *rom4* locus experienced a large expansion, giving rise to a gene family of five paralogous members. Thus, an increased number of serine proteinases of ROM4 can be observed between *B. bovis*, encoding five ROM4 vs. *T. annulata*, encoding two ROM4 paralogs [67,69]. While ROM6, located in the mitochondrion, is ubiquitous in eukaryotic organisms, ROM4, 7, and 8 are exclusively present in Aconoidasida. All piroplasmid ROM8 are predicted to lack serine protease activity, and are thus pseudoproteases, with a still unknown functional role [44].

Noteworthy, in the present study we have been able to identify an additional distinct S54 rhomboid proteinase type referred to as derlin in *T. annulata* (XP_952590) and *B. bovis* (XP_001612151 and XP_001611988), which were not reported before [67]. Derlins (degradation in endoplasmic reticulum protein) mediate the retrotranslocation of misfolded luminal proteins from the endoplasmic reticulum to the cytosol and represent a fifth group of ROM proteinases in piroplasmids [70]. BLAST search suggests that piroplasmids of all six phylogenetic lineages each encode two derlin proteins; however, an exception is *T. annulata,* where only a single derlin proteinase could be identified (Table S1, data not shown).

Proteinase families, supposed to have originated in bacteria after the advent of LUCA, include the C1 family [57]. These cysteine proteases are present in eukaryotes and bacteria but are absent from most archaea, and are thus supposed to have been transferred by endosymbiotic bacteria, such as those that gave origin to the protomitochondrion, to an ancestral eukaryotic organism [57]. The C1 family belongs to clan CA, whose members are usually referred to as C1A proteinases. Molecular phylogeny and classification of identified proteinases shows that the C1A type has suffered a significant expansion in *T. annulate,* as compared to *B. bovis*, with 13 and four representatives, respectively (Figure 1; Table 2) [71,72,73]. Recently, we have reported similar expansions of the C1A proteinase family in other *Theileria* s.s. species as well, with 13 members in *T. parva* and 15 in *T. orientalis*, while *Babesia* s.s. species *B. ovis* showed four, and *B. bigemina* showed five family members. Notably, *C. felis* and *T. equi,* that have, like *Theileria* s.s., a schizont stage in their life cycle, display also a moderate to extreme expansion of the C1A family (eight and 14 representatives in *C. felis* and *T. equi*, respectively) [73]. 

Furthermore, we showed by molecular phylogenetic analysis that the family C1A can be further subdivided into eight groups (C1A group 1 to 8). The phylogenetic lineage of *Theileria* s.s. was found to encode additional C1A proteinase groups 2 and 7, compared to *B. bovis*, and also a highly expanded number of C1A proteinases in group 8, comprising a single proteinase in *B. bovis* vs. seven paralogs in *T. annulata*. Thus, characteristic profiles of C1A proteinases of groups 1 to 9 were found to be associated with each of the six phylogenetic lineages of piroplasmids [73]. The marked difference between *Theileria* s.s. and *Babesia* s.s., which belong to phylogenetic Clades V and VI [8,9], respectively, suggests that C1A proteinase genes represent taxonomic markers for these groups. They furthermore substantiate the hypothesis that C1A expansion and life cycle characteristics of *Theileria* s.s., such as the development of a schizont stage, might be evolutionarily associated, although an involvement in host specificity and/or mode of transmission cannot be ruled out [73]. 

Falcipain-2, a prominent papain-like C1A protease of *P. falciparum*, has been shown to fulfill a dual function of hemoglobin degradation and cleavage of the erythrocyte membrane proteins ankyrin and protein 4.1. The latter activity facilitates the egress of the parasite from the erythrocyte and the continuation of its asexual reproduction in its mammalian host [74]. Falcipain-2 orthologs have been described and characterized in *B. bovis* (bovipain-2, XP_001610695), *B. bigemina* (babesipain-1, XP_012769730), and *B. ovis* (ovipain-2, ALJ75576). Consistent with a conserved function, these proteins were detected, as in the case of falcipain-2, inside intraerythrocytic merozoites as well as released to the cytoplasm of infected erythrocytes [72,75,76]. 

Due to their predicted functional relevance, different approaches have been applied to identify kinetics parameters, substrate specificity, and/or inhibitors of *Babesia* spp. C1A proteases, including producing enzymatically active recombinant proteins and in silico modeling and prediction analyses [75,77,78,79]. 

Given the conserved 3D structure of the active site of falcipain-2 and its babesial homologs [74], the identification of inhibitors for one of these enzymes may lead to the design of babesicide drugs of an ample spectrum. Moreover, identification of inhibitors of babesial papain-like enzymes may be tried as anti-malarial compounds. 

Antibodies against babesial C1A proteases of *B. ovis* and *B. bovis* significantly hamper the in vitro growth of these parasites in sheep or bovine erythrocytes, respectively, highlighting their functional relevance in the asexual intraerythrocytic phase of the babesial life cycle [76,79]. Additionally, cysteine proteases might be relevant for host-pathogen interactions at the tick level. In the tick, parasites undergo transformations, invasion, and egress to and from different tissues, and metabolic processes in which proteases are likely to participate. Notably, parasite invasion can represent a hazard for ticks, which in turn have developed a number of defense mechanisms to prevent a parasite overburden in their tissues and organs [11,80]. Tick cysteine protease inhibitors or cystatins are likely to be part of this anti-piroplasmid defense arsenal, since recombinant forms of *Haemaphysalis longicornis* cystatin-2 and *Rhipicephalus microplus* cystatin-1b, with proved inhibitory activity on cysteine proteases, impaired in vitro growth of *B. bovis* [81,82]. Additionally, *R. haemaphysaloides* ticks fed on *B. microti*-infected mice produced significantly higher levels of transcripts of cystatins than ticks fed on non-infected mice, supporting the notion of the participation of cystatins in parasite-tick relationships [83]. 

The ubiquitin-proteasome protein degradation system (UPS) is a multicomponent protein complex that takes care of degrading unwanted or misfolded proteins in all living organisms [84]. Network analysis of protein interactions carried out in *P. falciparum* allowed to identify that the following proteases are associated to the UPS: eleven threonine proteases of the T1 family, two proteases of the C12 ubiquitin C-terminal hydrolase family, and six proteases in the C19 ubiquitin-specific protease family [32]. Of these, the T1 and C19 families are present in *B. bovis* (T1, *n* = 14; C19, *n* = 6) and *T. annulata* (T1, *n* = 15; C19, *n* = 7), whereas a single C12 proteinase is only found in *B. bovis*. Interestingly, proteasome inhibitors were shown to hamper the in vitro and in vivo growth of *B. divergens* and *B. microti* in mice, respectively, highlighting the potential usefulness of the proteasome and its components as chemotherapeutic targets against *Babesia* spp. infections [85,86]. 

Proteinases can bear multiple proteinase and/or nonproteinase homolog domains, which may allow for the positive or negative modulation of proteinase activity, but also create novel regulatory or binding functions. Altogether, six proteinases composed of dual proteinase domains M16-M16, M23-M23, and S9-S9 were identified in *B. bovis*, but only two proteinases bearing M16-M16 domains were identified in *T. annulata*. Of these, a single dual proteinase M16-M16 in *B. bovis* (BBOV_IV001260) and in *T. annulata* (TA19130) were found to have a functional and a non-functional proteinase domain, of which the latter possibly exerts a modulatory function. All remaining dual-domain proteinases are composed of two nonproteinase homolog domains, suggesting they exert a regulatory or structural function, or modulate degradome activity. The substantial increased number of proteinases with dual non-functional domains in *B. bovis* might have functional implications in this species.

Altogether, 27 of the 133 proteinases of *B. bovis* and 36 of the 132 proteinases of *T. annulata* are secreted into the extracellular matrix or are membrane-associated. Membrane topology or cellular localization allow for predicting to which family a proteinase or nonproteinase homolog belongs and is associated with characteristic proteinase functions. Extracellular secretion is characteristic for A1 aspartic proteinases, single pass transmembrane proteinases seem to be confined to C1A and C13 cysteine proteinases and nonproteinase homologs, whereas multitopic proteinases or nonproteinase homologs typically belong to metallo (M41, M48, and M79) and serine proteinases and nonproteinase homologs (S26 and S54). Finally, an exclusive intracellular localization is observed for threonine proteinases (T1). Thus, these considerations allow to observe that the expansion of C1A proteinases in *T. annulata* and of S54 proteinases in *B. bovis* results in a significantly increased number of single pass membrane-associated proteinases in the former and an increased number of multitopic membrane-associated proteinases in the latter piroplasmid species. This suggests profound differences on the parasite–host interface of both species due to the corresponding substantial changes in the repertoire of C1A and S54 proteinases and nonproteinase homologs at the parasite surface.

The MEROPS database lists 271 families of proteolytic enzymes, which are defined by sequence comparison. The number of protease families varies for different phyla. For example, while only one protease family has been described for Bryozoa, some phyla of bacteria contain more than 150 different proteinase families, and 126 families have been described for Chordata. Of the 75 proteinase families described for the phylum Apicomplexa, proteinases of *B. bovis* and *T. annulata* can be allocated into 40 and 38 different families, respectively [57]. This difference is due to the presence of one proteinase of the S8 and one of the C12 families in *B. bovis*, which are both not found in *T. annulata.*

The S8 proteinase family is represented by a single serine endopeptidase subtilisin. Orthologs to *B. bovis* subtilisin (XP_001610126) were identified by BLAST searches in *B. ovis* (GFE54895), *B. ovata* (XP_028866959), *Babesia* sp. Xinjiang (XP_028872214), *B. divergens* (ABF60140), and *Babesia caballi* (GIX63735), suggesting that this S8 proteinase is characteristic for *Babesia* s.s. parasites. Supporting this notion, an ortholog of subtilisin is not encoded in the genome of *B. microti*, a representative of *Babesia* s.l. [56]. However, an ortholog was also found in a single parasite of *Theileria* s.s., *T. orientalis* (XP_009690597). Since S8 subtilisin is an ancient proteinase that was already present in LUCA, it is assumed that it has been lost in the other *Theileria* s.s. species and in *B. microti*., *B. divergens* subtilisin has been found to be localized in dense granules and released to the culture supernatant. Antibodies against a recombinant form of this protein blocked the erythrocyte invasion by the parasite, suggesting an important role for subtilisin in the life cycle of *B. divergens* [87]. It remains to be studied whether this role is conserved among remaining *Babesia* s.s. species and whether this protein is also relevant for the invasion process of *T. orientalis*. In this context, it should be noted that the invasion mechanism of *Theileria* s.s. parasites is profoundly distinct from that of *Babesia* s.s. [21].

Proteinases of the family C12 have emerged with the advent of eukaryotes [57]. Interestingly a representative of family C12 is present in all species of *Babesia* s.s. (with the exception of *B. divergens*) and also in *B. microti*, but not in *Theileria* s.s., *Theileria equi,* or *C. felis* (data not shown).

Notably, a proteinase annotated as signal peptide peptidase belonging to the aspartic proteinase family A22 has been identified in *B. microti* but found to be absent in both, *B. bovis* and *T. annulata* [56]. Proteinases of the family A22 have been reported to process intramembrane proteins and are present in the protozoans *P. falciparum* and *T. gondii* [57].

The present study also identified the repertoire of ancillary domains of both degradomes and assigned them to proteinases and nonproteinase homologs. Ancillary domains have highly diverse functional attributes. They may function as proteinase inhibitor domains, allosteric modulators, or exosite, mediate attachment to cellular structures, such as the cytoskeleton or membranes, and be involved in the ubiquitin/proteasome pathway. Commonly, ancillary domains have been functionally characterized in higher model organisms, and they might be involved in different functional contexts in evolutionary far distant piroplasmids. Functional differences between degradomes are thought to be reflected in differences in the composition of classes and families of proteinases, the presence of active proteinases vs. nonproteinase homologs, and orthologous pairs vs. species-specific proteinases. In contrast, the regulatory modulation of ancillary domains on degradome function is possibly underestimated. Substantial differences in the function and complexity of the degradome are most likely due to ancillary domains that control and modulate proteinase activity, mediate transport of proteinases to specific cellular locations, and are critical for the establishment of functional relevant protein-associated networks of proteinases. These proteinase-associated networks mediate cellular processes, such as information storage and processing, the ubiquitin-proteasome system, stress response, signal transduction, metabolism, as well as parasite movement, invasion, and egress [32].

Functional studies are lacking for most piroplasmid proteinase families. However, information gathered for *P. falciparum* can be used to infer some biological roles. For example, proteinases belonging to the S14 and S16, as well as M17 and M24 families, have been shown to be involved in heat shock responses that take place when this parasite passes from its mosquito vector to the human host; and, additionally, when malaria patients experience periodic rises in body temperature [32]. The corresponding proteinase families are present in *B. bovis* and *T. annulata*, and may have a similar function in these parasites, which also need to adapt to sudden temperature changes, upon passage from the tick vector to the mammalian host, and during hyperthermia of the mammalian hosts in the acute infection phase.

Some proteinases have been described as virulence factors for different protozoan parasites, including the aspartic protease plasmepsin 4 of *Plasmodium berghei* and the cysteine protease cruzipain of *Trypanosoma cruzi* [88,89]. In addition, increased proteinase activity in *B. bovis* virulent, as compared to avirulent strains, led to the hypothesis that proteinases are virulent determinants in this parasite [90,91]. However, comparative genomic and transcriptional studies carried out between a parental *B. bovis* virulent and the derived attenuated strain showed no changes in sequence and quantitative expression of any of the 66 tested genes encoding for functional proteinases [55]. Thus, although activity of different proteinases is likely required to establish infection in the mammalian host, as inferred from inhibitor and seroneutralization studies, changes in the nucleotide sequence and/or transcription levels of proteinase-encoding genes are not associated with the virulent/attenuated phenotype. 

In the case of *T. annulata*, studies with inhibitors suggested that its capacity to induce a metastatic status in host cells is associated with metalloprotease enzymatic activity [92]. Notably, virulence-attenuated strains of both *T. annulata* and the related species *T. lestoquardi* display reductions in metalloprotease gene expression and enzymatic activity [93,94,95]. 

The *T. annulata* C1A nonproteinase homolog XP_952571/TA11565, which is ortholog to *B. bovis* XP_001612131/BBOV_III010070, has been recently characterized, and its interactions with bovine proteins demonstrated by two-hybrid yeast experiments. It has been observed that this cysteine nonproteinase homolog interacts with two host proteins (CRBN, *Bos taurus* cereblon transcript variant X2, and Ppp4C, *Bos indicus* protein phosphatase 4 catalytic subunit), involved in a number of cellular processes connected to signaling pathways and cell proliferation [96]. This type of approach could be useful as an initial step to study the activity of other nonproteinase homologs of bovine piroplasmids, which should then be followed by experimental confirmation.

## 4. Materials and Methods

### 4.1. Mining of the Proteinase Repertoires

The genomes of *B. bovis* T2Bo and *T. annulata* Ankara C9 were used as primary sources to download the complete predicted proteomes, as accessible in Genbank and geneDB, respectively [6,7].

**Figure 3:**
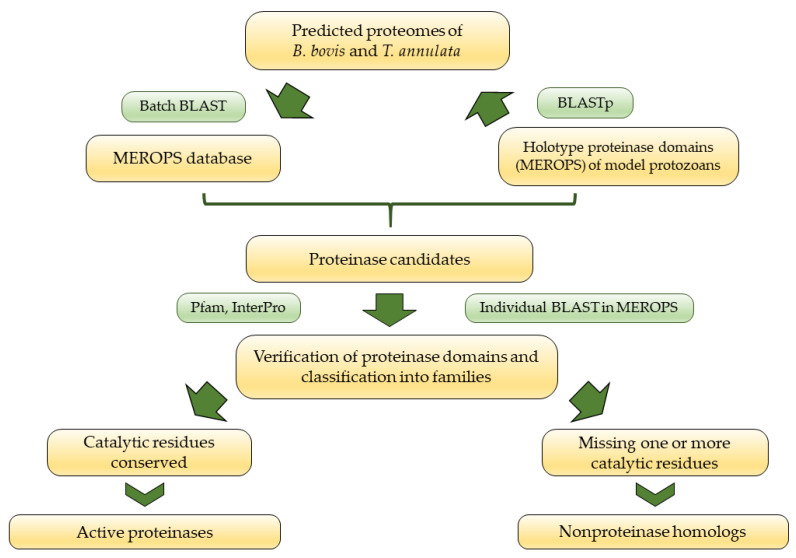
Bioinformatic pipeline used to identify active proteinases and nonproteinase homologs.

Proteinase identification and definition were performed in both genomes by a dual mining approach using tools available in the MEROPS database [97]. On the one hand, the complete proteome of each species was screened using the Batch BLAST tool applying an E-value threshold of 10^−10^ and 10^−4^ for the identification of the putative functional and non-functional proteinases, respectively. On the other hand, the proteinase domain of each family model proteinase of protozoans, as reported in the MEROPS database, was selected for a BLASTp analysis of the reference proteome of *B. bovis* and *T. annulata* in the Genbank. Each identified proteinase was subsequently used as query in an individual BLAST search against the MEROPS database and verified proteinase domains were confirmed by InterProScan and Pfam [98,99]. Proteinases identified and confirmed were classified into the five different protease groups, families and clans, and determined as functional or non-functional, based on presence vs. partial or complete absence of catalytic residues in the active site, respectively (Figure 3).

### 4.2. Bioinformatic and Phylogenetic Analyses

The presence of signal peptides, transmembrane regions and topology, and extracellular or intracellular location was verified using DeepTMHMM, which substantially improves prediction against previously available algorithms [100]. The presence of additional nonproteinase and/or ancillary domains in defined functional and/or non-functional proteinases was checked by searching the Pfam database, using an E-value threshold of 0.05.

Ortholog pairs were defined using a BLASTp bidirectional best hit (BBH) approach and by phylogenetic analyses of conserved amino acid domains [66,101].

Alignment of amino acid sequences of proteinase domains, including active site regions, was carried out using ClustalW. In case a proteinase comprised two proteinase domains, each of the domains was considered independently in the alignment. Evolutionary distances were estimated using the Dayhoff matrix. A phylogenetic tree was constructed based on the neighbor-joining algorithm with 1000 bootstrap replicates using the MEGA7 program, as follows [102]. In a first step, a basic tree was built using one proteinase sequence from each family, and then, in a second step, individual trees previously built for each family were joined upon this basic tree. 

## 5. Conclusions

The objective of this study is to describe and identify differences between the degradomes of the bovine piroplasmid pathogens *B. bovis* and *T. annulata*. Altogether, 25 species-specific proteinases (seven proteinases vs. 18 nonproteinase homologs) could be identified in *B. bovis*, and 24 (14 proteinases vs. 10 nonproteinase homologs) in *T. annulata.* Species-specific proteinases and nonproteinase homologs belong mainly to serine rhomboid proteinases in *B. bovis* (*n* = 18 vs. *n* = 5), but predominantly to cysteine proteinases in *T. annulata* (*n* = 12 vs. *n* = 2). Among active species-specific proteinases, mainly an expansion of C1A proteinase paralogs in *T. annulata* and of S54 proteinase paralogs in *B. bovis* contribute to this dissimilarity generated by successive gene duplication events. The latter notion is supported by observation of the conserved synteny of corresponding gene families. The increased number of single-pass C1A membrane proteinases in *T. annulata* and S54 multi-pass membrane proteinases in *B. bovis* potentially results in substantial structural differences of the surface membrane between both piroplasmid species. Notably, only *B. bovis* encodes a serine proteinase of the family S8 (subtilisin-like proteinase) and the family C12 (ubiquitin carboxy-terminal hydrolase). Furthermore, exclusively in *B. bovis,* an additional nonproteinase homolog with dual M23-M23 domains and three with dual S9-S9 domains were identified. Finally, a large number of species-specific ancillary domains were detected in both *B. bovis* and *T. annulata*. We hypothesize that this results in substantial differences in proteinase interaction with other proteins, and changes in the activity and regulation of each degradome. Some of the differences described between *B. bovis* and *T. annulata* are also observed to varying degrees between species belonging to different phylogenetic lineages of piroplasmids (clades I to VI), and may be considered evolutionary adaptations to different host environments and life histories.

The presented degradome analysis will aid in the selection of candidate proteinases for functional studies or as potential drug targets, and will allow revealing evolutionary patterns between degradomes that are associated with diverse phylogenetic lineages of piroplasmids.

## Figures and Tables

**Table 1:** Composition of the degradomes of *Babesia bovis* and *Theileria annulata*.

	Proteinases and Nonproteinase Homologs						
Proteinase Repertoire	Total	Proteinases vs. Nonproteinase Homologs ^a^	Catalytic Type				
			Aspartic	Cysteine	Metallo	Serine	Threonine
*B. bovis*	133	82/51	6	26	36	51	14
*T. annulata*	132	80/52	8	36	35	38	15

^a^ Number of functional proteinases vs. nonproteinase homologs; all proteinases and nonproteinase homologs listed in Table S1 are considered in this table.

**Table 2:** Orthologous pairs and species-specific proteinases and nonproteinase homologs of *Babesia bovis* and *Theileria annulata*.

		Proteinases and Nonproteinase Homologs ^a^					
	Total	Proteinases vs. Nonproteinase Homologs	Catalytic Type				
			Aspartic	Cysteine	Metallo	Serine	Threonine
(a) Orthologous proteinases							
Orthologous pairs	108		5	24	32	33	14
Proteinases vs. non proteinase homologs	108	54/48/6 ^a^	5/0	18/6	15/17	11/22	5/9
(b) Species-specific proteinases							
*B. bovis*-specific	25	7/18	1	2	4	18	0
*T. annulata*-specific	24	14/10	3	12	3	5	1

^a^ All proteinases and nonproteinase homologs listed in Table S1 are considered in this table.

**Table 3:** Proteinases with dual proteinase and nonproteinase homolog domains.

Dual Domain Composition	Gene ID	Upstream Domain ^a^	Downstream Domain ^b^
M16-M16	BBOV_III003850/TA11975	a ^nf^	b ^nf^
	BBOV_IV001260/TA19130	a ^f^	b ^nf^
M23-M23	BBOV_II001730	a ^nf^	b ^nf^
S9-S9	BBOV_III011180	a ^nf^	b ^nf^
	BBOV_IV003330	a ^nf^	b ^nf^
	BBOV_II003080	a ^nf^	b ^nf^

^a^ upstream or N-terminal domain a; ^b^ downstream or C-terminal domain b; ^nf^ domain of nonproteinase homolog; ^f^ functional proteinase domain.

**Table 4:** Species-specific functional proteinases of *Babesia bovis* and *Theileria annulata*.

Catalytic Type	Family ^a^	*B. bovis*		*T. annulata*	
		Gene ID ^b^	*n*	Gene ID ^b^	*n*
Aspartic	A1	BBOV_IV010360	1	-	0
Cysteine	C1	-	0	TA03720; TA03735; TA03740;TA03745; TA03750; TA10955; TA15660; TA15715	8
	C12	BBOV_III010630	1	-	0
	C19	-	0	TA16070	1
	C48	-	0	TA11130	1
	C86	-	0	TA12030	1
	C88	-	0	TA14855	1
	C97	BBOV_II001800	1	-	0
Metallo	M16	-	0	TA12175	1
Serine	S8	BBOV_II006080	1	-	0
	S9	BBOV_IV005690	1	-	0
	S33	-	0	TA18005	1
	S54	BBOV_II005930; BBOV_II005940; BBOV_II006070; BBOV_III008600	4	-	1
Threonine	T1	-	0	TA15900	1

^a^ Proteinase family designation according to MEROPS; ^b^ Accession numbers of gene loci of *B. bovis* and *T. annulata* correspond to those from GenBank and piroplasmaDB, respectively.

## Data Availability

Data is contained within the article and supplementary material.

## Supplementary Materials

The following supporting information can be downloaded at: https://www.mdpi.com/article/10.3390/pathogens12020237/s1, Figure S1: Comprehensive list of proteinases and nonproteinase homolog repertoire of *Babesia bovis* and *Theileria annulata;* Table S1: Classification of proteinases and nonproteinase homologs of *Babesia bovis* and *Theileria annulata* into catalytic type, clan, and family; Table S2: Secreted and membrane-associated proteinases and nonproteinase homologs of *Babesia bovis* and *Theileria annulata* degradomes.

## References

[1] Zhang Z. (1997). A general review on the prevention and treatment of Theileria annulata in China. Vet. Parasitol..

[2] Inci A., Ica A., Yildirim A., Vatansever Z., Cakmak A., Albasan H., Cam Y., Atasever A., Sariozkan S., Duzlu O. (2007). Economical impact of tropical theileriosis in the Cappadocia region of Turkey. Parasitol. Res..

[3] El Hussein A.M., Hassan S.M., Salih D. (2012). Current situation of tropical theileriosis in the Sudan. Parasitol. Res..

[4] Florin-Christensen M., Suarez C.E., Rodriguez A.E., Flores D.A., Schnittger L. (2014). Vaccines against bovine babesiosis: Where we are now and possible roads ahead. Parasitology.

[5] Florin-Christensen M., Schnittger L., Bastos R.G., Rathinasamy V.A., Cooke B.M., Alzan H.F., Suarez C.E. (2021). Pursuing effective vaccines against cattle diseases caused by apicomplexan protozoa. CAB Rev..

[6] Pain A., Renauld H., Berriman M., Murphy L., Yeats C.A., Weir W., Kerhornou A., Aslett M., Bishop R., Bouchier C. (2005). Genome of the Host-Cell Transforming Parasite *Theileria annulata* Compared with *T. Parva*. Science.

[7] Brayton K.A., Lau A.O.T., Herndon D.R., Hannick L., Kappmeyer L.S., Berens S.J., Bidwell S.L., Brown W.C., Crabtree J., Fadrosh D. (2007). Genome Sequence of Babesia bovis and Comparative Analysis of Apicomplexan Hemoprotozoa. PLOS Pathog..

[8] Schnittger L., Rodriguez A.E., Florin-Christensen M., Morrison D.A. (2012). Babesia: A world emerging. Infect. Genet. Evol..

[9] Schnittger L., Ganzinelli S., Bhoora R., Omondi D., Nijhof A.M., Florin-Christensen M. (2022). The Piroplasmida Babesia, Cytauxzoon, and Theileria in farm and companion animals: Species compilation, molecular phylogeny, and evolutionary insights. Parasitol. Res..

[10] Jalovecka M., Sojka D., Ascencio M., Schnittger L. (2019). Babesia Life Cycle—When Phylogeny Meets Biology. Trends Parasitol..

[11] Florin-Christensen M., Schnittger L. (2009). Piroplasmids and ticks: A long-lasting intimate relationship. Front. Biosci..

[12] Ganzinelli S., Rodriguez A.E., Schnittger L., Florin-Christensen M., Florin-Christensen M., Schnittger L. (2018). Parasitic Protozoa of Farm Animals and Pets.

[13] Jalovecka M., Hajdusek O., Sojka D., Kopacek P., Malandrin L. (2018). The Complexity of Piroplasms Life Cycles. Front. Cell. Infect. Microbiol..

[14] Gray J.S., Estrada-Peña A., Zintl A. (2019). Vectors of Babesiosis. Annu. Rev. Entomol..

[15] Hunfeld K.-P., Hildebrandt A., Gray J.S. (2008). Babesiosis: Recent insights into an ancient disease. Int. J. Parasitol..

[16] Dolan T.T. (1989). Theileriasis: A comprehensive review. Rev. Sci. Tech. Off. Int. Des Epizoot..

[17] Kiara H., Steinaa L., Vishvanath N., Svitek N., Florin-Christensen M., Schnittger L. (2018). Babesia in domestic ruminants. Parasitic Protozoa of Farm Animals and Pets.

[18] Spooner R.L., Innes E.A., Glass E.J., Brown C.G. (1989). Theileria annulata and T. parva infect and transform different bovine mon-onuclear cells. Immunology.

[19] Tajeri S., Haidar M., Sakura T., Langsley G. (2021). Interaction between transforming *Theileria* parasites and their host bovine leukocytes. Mol. Microbiol..

[20] Sivakumar T., Hayashida K., Sugimoto C., Yokoyama N. (2014). Evolution and genetic diversity of Theileria. Infect. Genet. Evol..

[21] Shaw M.K. (2003). Cell invasion by Theileria sporozoites. Trends Parasitol..

[22] Rosenthal P., Sijwali P., Singh A., Shenai B. (2002). Cysteine Proteases of Malaria Parasites: Targets for Chemotherapy. Curr. Pharm. Des..

[23] Yang G., Li J., Zhang X., Zhao Q., Liu Q., Gong P. (2008). Eimeria tenella: Construction of a recombinant fowlpox virus expressing rhomboid gene and its protective efficacy against homologous infection. Exp. Parasitol..

[24] Fedeli C.E.C., Ferreira J.H.L., Mussalem J.S., Longo-Maugéri I.M., Gentil L.G., dos Santos M.R.M., Katz S., Barbiéri C.L. (2010). Partial protective responses induced by a recombinant cysteine proteinase from Leishmania (Leishmania) amazonensis in a murine model of cutaneous leishmaniasis. Exp. Parasitol..

[25] Cai H., Kuang R., Gu J., Wang Y. (2011). Proteases in Malaria Parasites—A Phylogenomic Perspective. Curr. Genom..

[26] Pandey K.C. (2011). Centenary celebrations article: Cysteine proteases of human malaria parasites. J. Parasit. Dis..

[27] Li J., Zheng J., Gong P., Zhang X. (2012). Efficacy of Eimeria tenella rhomboid-like protein as a subunit vaccine in protective immunity against homologous challenge. Parasitol. Res..

[28] Silva-Almeida M., Pereira B.A.S., Ribeiro-Guimarães M.L., Alves C.R. (2012). Proteinases as virulence factors in *Leishmania* spp. infection in mammals. Parasit. Vectors.

[29] Zhang N.-Z., Xu Y., Wang M., Petersen E., Chen J., Huang S.-Y., Zhu X.-Q. (2015). Protective efficacy of two novel DNA vaccines expressing *Toxoplasma gondii* rhomboid 4 and rhomboid 5 proteins against acute and chronic toxoplasmosis in mice. Expert Rev. Vaccines.

[30] Rawlings N.D. (2019). Twenty-five years of nomenclature and classification of proteolytic enzymes. Biochim. Biophys. Acta Proteins Proteom..

[31] Klemba M., Goldberg D.E. (2002). Biological Roles of Proteases in Parasitic Protozoa. Annu. Rev. Biochem..

[32] Lilburn T.G., Cai H., Zhou Z., Wang Y. (2011). Protease-associated cellular networks in malaria parasite Plasmodium falciparum. BMC Genom..

[33] Sojka D., Šnebergerová P., Robbertse L. (2021). Protease Inhibition—An Established Strategy to Combat Infectious Diseases. Int. J. Mol. Sci..

[34] Harris P.K., Yeoh S., Dluzewski A.R., A O’Donnell R., Withers-Martinez C., Hackett F., Bannister L.H., Mitchell G.H., Blackman M.J. (2005). Molecular Identification of a Malaria Merozoite Surface Sheddase. PLOS Pathog..

[35] Yeoh S., O’Donnell R.A., Koussis K., Dluzewski A.R., Ansell K.H., Osborne S.A., Hackett F., Withers-Martinez C., Mitchell G.H., Bannister L.H. (2007). Subcellular Discharge of a Serine Protease Mediates Release of Invasive Malaria Parasites from Host Erythrocytes. Cell.

[36] Koussis K., Withers-Martinez C., Yeoh S., Child M., Hackett F., Knuepfer E., Juliano L., Woehlbier U., Bujard H., Blackman M.J. (2009). A multifunctional serine protease primes the malaria parasite for red blood cell invasion. EMBO J..

[37] Šnebergerová P., Bartošová-Sojková P., Jalovecká M., Sojka D. (2021). Plasmepsin-like Aspartyl Proteases in *Babesia*. Pathogens.

[38] Sim B.K.L., Chitnis C.E., Wasniowska K., Hadley T.J., Miller L.H. (1994). Receptor and Ligand Domains for Invasion of Erythrocytes by *Plasmodium falciparum*. Science.

[39] Baker R.P., Wijetilaka R., Urban S. (2006). Two Plasmodium Rhomboid Proteases Preferentially Cleave Different Adhesins Implicated in All Invasive Stages of Malaria. PLOS Pathog..

[40] O’Donnell R.A., Hackett F., Howell S.A., Treeck M., Struck N., Krnajski Z., Withers-Martinez C., Gilberger T.-W., Blackman M.J. (2006). Intramembrane proteolysis mediates shedding of a key adhesin during erythrocyte invasion by the malaria parasite. J. Cell Biol..

[41] Urban S. (2009). Making the cut: Central roles of intramembrane proteolysis in pathogenic microorganisms. Nat. Rev. Microbiol..

[42] Strisovsky K. (2016). Rhomboid protease inhibitors: Emerging tools and future therapeutics. Semin. Cell Dev. Biol..

[43] Tichá A., Collis B., Strisovsky K. (2018). The Rhomboid Superfamily: Structural Mechanisms and Chemical Biology Opportunities. Trends Biochem. Sci..

[44] Gandhi S., Baker R.P., Cho S., Stanchev S., Strisovsky K., Urban S. (2020). Designed Parasite-Selective Rhomboid Inhibitors Block Invasion and Clear Blood-Stage Malaria. Cell Chem. Biol..

[45] Francis S.E., Sullivan D.J., Goldberg D.E. (1997). Hemoglobin metabolism in the malaria parasite *Plasmodium falciparum*. Annu. Rev. Microbiol..

[46] Coombs G.H., Goldberg D.E., Klemba M., Berry C., Kay J., Mottram J. (2001). Aspartic proteases of Plasmodium falciparum and other parasitic protozoa as drug targets. Trends Parasitol..

[47] Hanspal M., Dua M., Takakuwa Y., Chishti A.H., Mizuno A. (2002). Plasmodium falciparum cysteine protease falcipain-2 cleaves erythrocyte membrane skeletal proteins at late stages of parasite development. Blood.

[48] Liu J., Gluzman I.Y., Drew M.E., Goldberg D.E. (2004). The Role of Plasmodium falciparum Food Vacuole Plasmepsins. J. Biol. Chem..

[49] Sijwali P.S., Rosenthal P.J. (2004). Gene disruption confirms a critical role for the cysteine protease falcipain-2 in hemoglobin hydrolysis by *Plasmodium falciparum*. Proc. Natl. Acad. Sci. USA.

[50] Arastu-Kapur S., Ponder E.L., Fonović U.P., Yeoh S., Yuan F., Fonovic M., Grainger M., Phillips C.I., Powers J.C., Bogyo M. (2008). Identification of proteases that regulate erythrocyte rupture by the malaria parasite Plasmodium falciparum. Nat. Chem. Biol..

[51] Okubo K., Yokoyama N., Govind Y., Alhassan A., Igarashi I. (2007). Babesia bovis: Effects of cysteine protease inhibitors on in vitro growth. Exp. Parasitol..

[52] Aboge G.O., Cao S., Terkawi M.A., Masatani T., Goo Y., AbouLaila M., Nishikawa Y., Igarashi I., Suzuki H., Xuan X. (2015). Molecular Characterization of *Babesia bovis* M17 Leucine Aminopeptidase and Inhibition of *Babesia* Growth by Bestatin. J. Parasitol..

[53] Munkhjargal T., Ishizaki T., Guswanto A., Takemae H., Yokoyama N., Igarashi I. (2016). Molecular and biochemical characterization of methionine aminopeptidase of Babesia bovis as a potent drug target. Vet. Parasitol..

[54] Sojka D., Jalovecká M., Perner J. (2022). Babesia, Theileria, Plasmodium and Hemoglobin. Microorganisms.

[55] Mesplet M., Palmer G.H., Pedroni M.J., Echaide I., Florin-Christensen M., Schnittger L., Lau A.O. (2011). Genome-wide analysis of peptidase content and expression in a virulent and attenuated Babesia bovis strain pair. Mol. Biochem. Parasitol..

[56] Florin-Christensen M., Wieser S.N., Suarez C.E., Schnittger L. (2021). In Silico Survey and Characterization of *Babesia microti* Functional and Non-Functional Proteases. Pathogens.

[57] Rawlings N.D., Bateman A. (2019). Origins of peptidases. Biochimie.

[58] Rawlings N.D., Barrett A.J., Finn R. (2015). Twenty years of the *MEROPS* database of proteolytic enzymes, their substrates and inhibitors. Nucleic Acids Res..

[59] López-Otín C., Bond J.S. (2008). Proteases: Multifunctional Enzymes in Life and Disease. J. Biol. Chem..

[60] E Todd A., A Orengo C., Thornton J.M. (2002). Sequence and Structural Differences between Enzyme and Nonenzyme Homologs. Structure.

[61] Pils B., Schultz J. (2004). Inactive Enzyme-homologues Find New Function in Regulatory Processes. J. Mol. Biol..

[62] Reynolds S.L., Fischer K. (2015). Pseudoproteases: Mechanisms and function. Biochem. J..

[63] Adrain C., Cavadas M. (2020). The complex life of rhomboid pseudoproteases. FEBS J..

[64] Mishra L., Funk C. (2021). The FtsHi Enzymes of *Arabidopsis thaliana*: Pseudo-Proteases with an Important Function. Int. J. Mol. Sci..

[65] Zupanič N., Počič J., Leonardi A., Šribar J., Kordiš D., Križaj I. (2022). Serine pseudoproteases in physiology and disease. FEBS J..

[66] Koonin E.V. (2005). Orthologs, Paralogs, and Evolutionary Genomics. Annu. Rev. Genet..

[67] Gallenti R., Poklepovich T., Florin-Christensen M., Schnittger L. (2021). The repertoire of serine rhomboid proteases of piroplasmids of importance to animal and human health. Int. J. Parasitol..

[68] Dowse T.J., Koussis K., Blackman M.J., Soldati-Favre D. (2008). Roles of Proteases during Invasion and Egress by Plasmodium and Toxoplasma. Subcell. Biochem..

[69] Gallenti R., Hussein H.E., Alzan H.F., Suarez C.E., Ueti M., Asurmendi S., Benitez D., Araujo F.R., Rolls P., Sibeko-Matjila K. (2022). Unraveling the Complexity of the Rhomboid Serine Protease 4 Family of *Babesia bovis* Using Bioinformatics and Experimental Studies. Pathogens.

[70] Nejatfard A., Wauer N., Bhaduri S., Conn A., Gourkanti S., Singh N., Kuo T., Kandel R., Amaro R.E., Neal S.E. (2021). Derlin rhomboid pseudoproteases employ substrate engagement and lipid distortion to enable the retrotranslocation of ERAD membrane substrates. Cell Rep..

[71] Sajid M., Blackman M.J., Doyle P., He C., Land K.M., Lobo C., Mackey Z., Ndao M., Reed S.L., Shiels B., Selzer P.M. (2009). Antipara-sitic and Antibacterial Drug Discovery: From Molecular Targets to Drug Candidates.

[72] Mesplet M., Echaide I., Dominguez M., Mosqueda J.J., E Suarez C., Schnittger L., Florin-Christensen M. (2010). Bovipain-2, the falcipain-2 ortholog, is expressed in intraerythrocytic stages of the tick-transmitted hemoparasite Babesia bovis. Parasit. Vectors.

[73] Ascencio M.E., Florin-Christensen M., Mamoun C.B., Weir W., Shiels B., Schnittger L. (2018). Cysteine Proteinase C1A Paralog Profiles Correspond with Phylogenetic Lineages of Pathogenic Piroplasmids. Vet. Sci..

[74] Rosenthal P.J. (2004). Cysteine proteases of malaria parasites. Int. J. Parasitol..

[75] Martins T.M., Rosário V.E.D., Domingos A. (2011). Identification of papain-like cysteine proteases from the bovine piroplasm Babesia bigemina and evolutionary relationship of piroplasms C1 family of cysteine proteases. Exp. Parasitol..

[76] Carletti T., Barreto C., Mesplet M., Mira A., Weir W., Shiels B., Oliva A.G., Schnittger L., Florin-Christensen M. (2016). Characterization of a papain-like cysteine protease essential for the survival of Babesia ovis merozoites. Ticks Tick-Borne Dis..

[77] Pérez B., Antunes S., Gonçalves L.M., Domingos A., Gomes J.R.B., Gomes P., Teixeira C. (2013). Toward the discovery of inhibitors of babesipain-1, a Babesia bigemina cysteine protease: In vitro evaluation, homology modeling and molecular docking studies. J. Comput. Aided Mol. Des..

[78] Meetei P.A., Rathore R.S., Prabhu N.P., Vindal V. (2016). Modeling of babesipain-1 and identification of natural and synthetic leads for bovine babesiosis drug development. J. Mol. Model..

[79] Lu S., Ascencio M.E., Torquato R.J., Florin-Christensen M., Tanaka A.S. (2020). Kinetic characterization of a novel cysteine peptidase from the protozoan Babesia bovis, a potential target for drug design. Biochimie.

[80] Sonenshine D.E., Macaluso K.R. (2017). Microbial Invasion vs. Tick Immune Regulation. Front. Cell. Infect. Microbiol..

[81] Zhou J., Ueda M., Umemiya R., Battsetseg B., Boldbaatar D., Xuan X., Fujisaki K. (2006). A secreted cystatin from the tick Haemaphysalis longicornis and its distinct expression patterns in relation to innate immunity. Insect Biochem. Mol. Biol..

[82] Lu S., da Rocha L.A., Torquato R.J., Jr I.D.S.V., Florin-Christensen M., Tanaka A.S. (2020). A novel type 1 cystatin involved in the regulation of Rhipicephalus microplus midgut cysteine proteases. Ticks Tick-Borne Dis..

[83] Wei N., Du Y., Lu J., Zhou Y., Cao J., Zhang H., Gong H., Zhou J. (2020). A cysteine protease of Babesia microti and its interaction with tick cystatins. Parasitol. Res..

[84] Hershko A., Ciechanover A. (1998). The ubiquitin system. Annu. Rev. Biochem..

[85] AbouLaila M., Nakamura K., Govind Y., Yokoyama N., Igarashi I. (2010). Evaluation of the in vitro growth-inhibitory effect of epoxomicin on Babesia parasites. Vet. Parasitol..

[86] Jalovecka M., Hartmann D., Miyamoto Y., Eckmann L., Hajdusek O., O’Donoghue A.J., Sojka D. (2018). Validation of Babesia proteasome as a drug target. Int. J. Parasitol. Drugs Drug Resist..

[87] Montero E., Rafiq S., Heck S., Lobo C.A. (2007). Inhibition of human erythrocyte invasion by Babesia divergens using serine protease inhibitors. Mol. Biochem. Parasitol..

[88] Spaccapelo R., Janse C.J., Caterbi S., Franke-Fayard B., Bonilla J.A., Syphard L.M., Di Cristina M., Dottorini T., Savarino A., Cassone A. (2010). Plasmepsin 4-Deficient Plasmodium berghei Are Virulence Attenuated and Induce Protective Immunity against Experimental Malaria. Am. J. Pathol..

[89] Francisco J.S., Barría I., Gutiérrez B., Neira I., Muñoz C., Sagua H., Araya J.E., Andrade J.C., Zailberger A., Catalán A. (2017). Decreased cruzipain and gp85/trans-sialidase family protein expression contributes to loss of Trypanosoma cruzi trypomastigote virulence. Microbes Infect..

[90] Wright I.G., Goodger B.V., Mahoney D.F. (1981). Virulent and Avirulent Strains ofBabesia bovis: The Relationship Between Parasite Protease Content and Pathophysiological Effect of the Strain. J. Protozool..

[91] Savon L.C., Alonso M., Rodriguez-Diego J., Blandino T. (1992). Determination of the protease activity in a Cuban strain of Babesia bovis. Rev. Elev. Med. Vet. Pays Trop..

[92] Adamson R.E., Hall F.R. (1996). Matrix metalloproteinases mediate the metastatic phenotype of *Theileria annulata*-transformed cells. Parasitology.

[93] Adamson R., Logan M., Kinnaird J., Langsley G., Hall R. (2000). Loss of matrix metalloproteinase 9 activity in Theileria annulata-attenuated cells is at the transcriptional level and is associated with differentially expressed AP-1 species. Mol. Biochem. Parasitol..

[94] Shkap V., Pipano E., Rasulov I., Azimov D., Savitsky I., Fish L., Krigel Y., Leibovitch B. (2003). Proteolytic enzyme activity and attenuation of virulence in Theileria annulata schizont-infected cells. Vet. Parasitol..

[95] Ali A.M., Beyer D., Bakheit M.A., Kullmann B., Salih D.A., Ahmed J.S., Seitzer U. (2008). Influence of subculturing on gene expression in a Theileria lestoquardi-infected cell line. Vaccine.

[96] Zhao S., Guan G., Liu J., Liu A., Li Y., Yin H., Luo J. (2017). Screening and identification of host proteins interacting with Theileria annulata cysteine proteinase (TaCP) by yeast-two-hybrid system. Parasit. Vectors.

[97] Rawlings N.D., Waller M., Barrett A.J., Bateman A. (2014). MEROPS: The database of proteolytic enzymes, their substrates and inhibitors. Nucleic Acids Res..

[98] Mistry J., Chuguransky S., Williams L., Qureshi M., Salazar G.A., Sonnhammer E.L.L., Tosatto S.C., Paladin L., Raj S., Richardson L.J. (2021). Pfam: The protein families database in 2021. Nucleic Acids Res..

[99] Paysan-Lafosse T., Blum M., Chuguransky S., Grego T., Pinto B.L., Salazar G.A., Bileschi M.L., Bork P., Bridge A., Col-well L. (2022). InterPro in 2022. Nucleic Acids Res..

[100] Hallgren J., Tsirigos K.D., Pedersen M.D., Armenteros J.J.A., Marcatili P., Nielsen H., Krogh A., Winther O. (2022). DeepTMHMM predicts alpha and beta transmembrane proteins using deep neural networks. bioRxiv.

[101] Tatusov R.L., Koonin E.V., Lipman D.J. (1997). A Genomic Perspective on Protein Families. Science.

[102] Tamura K., Peterson D., Peterson N., Stecher G., Nei M., Kumar S. (2011). MEGA5: Molecular Evolutionary Genetics Analysis Using Maximum Likelihood, Evolutionary Distance, and Maximum Parsimony Methods. Mol. Biol. Evol..

