# Supplementary material for: Comparative Degradome Analysis of the Bovine Piroplasmid Pathogens Babesia bovis and Theileria annulata
Source: Pathogens. 2023 Feb 2;12(2):237. doi: 10.3390/pathogens12020237 (PMC9965338; doi:10.3390/pathogens12020237)
Supplement: Supplementary file 1 [file pathogens-12-00237-s001.zip › Figure S1.pdf]

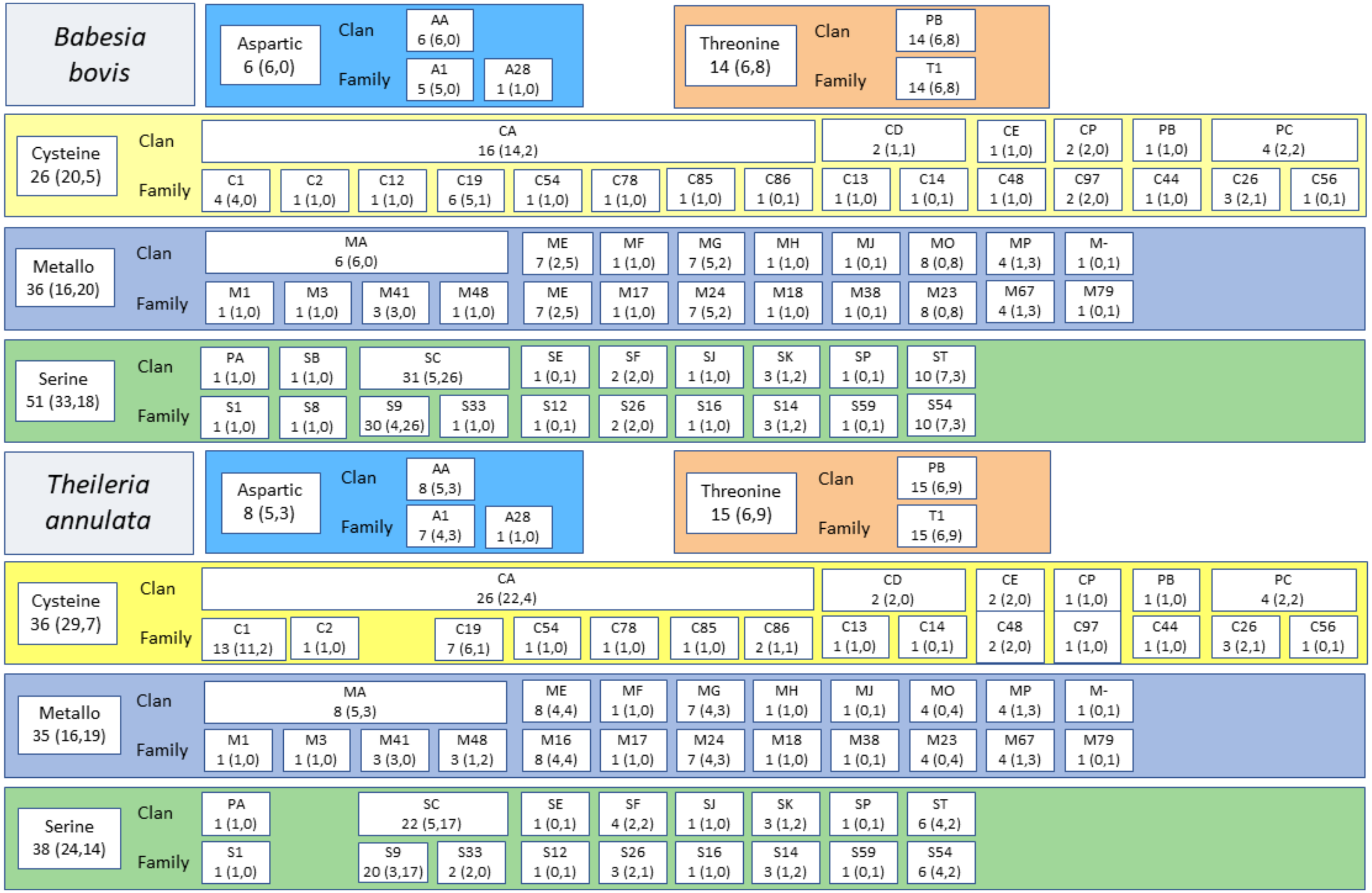

Figure S1. Classification of peptidases and nonpeptidase homologs of *B. bovis* and *T. annulata* into catalytic type, clan, and family according to MEROPS. The number of peptidases and nonproteinase homologs of each clan and family is indicated. The number of functional and nonfunctional peptidases is given in brackets. The metalloproteinase family M79 has been redesignated as glutamic proteinase family G05; however, this needs experimental confirmation.
