# Supplementary material for: Comparative Degradome Analysis of the Bovine Piroplasmid Pathogens Babesia bovis and Theileria annulata
Source: Pathogens. 2023 Feb 2;12(2):237. doi: 10.3390/pathogens12020237 (PMC9965338; doi:10.3390/pathogens12020237)
Supplement: Supplementary file 1 [file pathogens-12-00237-s001.zip › Table S2.pdf]

Table S2. Secreted and membrane-associated proteinases and nonproteinase homologs of *Babesia bovis* and *Theileria annulata* degradomes

	B. bovis			***T. annulata***		
**Proteinase family**	**Gene ID**	**signal peptide/ location**	**TMD**	**Gene ID**	**signal peptide/ location**	**TMD**
Aspartyl						
A1	BBOV_III003510	s/o		TA02510	s/o	
	BBOV_IV007890	s/o		TA02750	s/o	
	BBOV_IV009660	s/o		TA05735	s/o	
	BBOV_III001640	s/o		TA17685	s/o	
	BBOV_IV010360	s/o				
				TA03860 [nf]	o	
				TA05620 [nf]	o	
Cysteine						
C1	BBOV_I000540	o		TA15665	s/o	
	BBOV_II000170	s/o		TA04105	s/o	
	BBOV_III010070		iNo	TA11565		iNo
	BBOV_IV007730		iNo	TA03725		iNo
				TA03720		iNo
				TA03730 [nf]		iNo
				TA03735		iNo
				TA03740		iNo
				TA03745		iNo
				TA03750		iNo
				TA15660		iNo
				TA15715		iNo
				TA10955	s/o	
C13	BBOV_I000200	s	iCo	TA17465	o	
Metallo						
M23				TA20220 [nf]	o	
M41	BBOV_II000870		i2i	TA08725		iNo
	BBOV_III005230		i2i	TA13125		i2i
	BBOV_IV011870		i2i	TA16460		i1o
M48	BBOV_IV000310		o7i	TA15890		o7i
				TA15895 [nf]		o7i
				TA15870 [nf]		i6i
M79[b]	BBOV_III000740		i9o	TA18155		i9o

**Serine**					
S08	BBOV_II006080	s/o			
S9				TA17990 [nf]	i3i
S26	BBOV_III000270		i2i	TA18300	i2i
S54	BBOV_I003700		i2i	TA20680	i6i
	BBOV_II005950		i7o	TA13875	i7o
	BBOV_II006100		o6o	TA13905	i8i
	BBOV_III000530		i6i	TA18080	i6i
	BBOV_IV005790 [nf]		i5o	TA20965 [nf]	i7o
	BBOV_II005930		i6i		
	BBOV_II005940		i7o		
	BBOV_II006070	s	i7o		
	BBOV_III010270[a]		i6i	TA11460[a]	i6i
	BBOV_III008600[a]		i6i		

Proteinase and nonproteinase homolog families of aspartic proteinase (A), cysteine proteinase (C), metallo proteinase (M), and serine proteinase (S) are designated according to MEROPS; proteinases and/or nonproteinase homologs on the same line correspond to orthologs; the presence of a signal peptide (s) and extracellular location of proteinase (o) is indicated as s/o; single-pass proteins of type I are designated iCo and those of type II are designated iNo; when the transmembrane domain is located in the center of the protein sequence it is designated i1o; the topology of multitopic proteins is designated by indicating the intracellular (i) or extracellular location (o) of the N-terminal (left) or C-terminal (right) sequence regions and the number of transmembrane domains (TMDs); [nf]nonproteinase homolog with at least one incomplete active site of the catalytic region; TMD, transmembrane domain; [a]rhomboid proteinase belonging to the derlin family; [b]family M79 has been recently redesignated as glutamic proteinase family G5 in MEROPS
